# Genetic evidence of a northward range expansion in the eastern Bering Sea stock of Pacific cod

**DOI:** 10.1111/eva.12874

**Published:** 2019-10-18

**Authors:** Ingrid Spies, Kristen M. Gruenthal, Daniel P. Drinan, Anne B. Hollowed, Duane E. Stevenson, Carolyn M. Tarpey, Lorenz Hauser

**Affiliations:** ^1^ Alaska Fisheries Science Center National Oceanic Atmospheric Administration Seattle ‎Washington; ^2^ School of Aquatic and Fishery Sciences University of Washington Seattle ‎Washington; ^3^ Office of Applied Science Wisconsin Department of Natural Resources Wisconsin Cooperative Fishery Research Unit University of Wisconsin‐Stevens Point Stevens Point Wisconsin

**Keywords:** climate change, fisheries management, population dynamics, population genetics – empirical

## Abstract

Poleward species range shifts have been predicted to result from climate change, and many observations have confirmed such movement. Poleward shifts may represent a homogeneous shift in distribution, seasonal northward movement of specific populations, or colonization processes at the poleward edge of the distribution. The ecosystem of the Bering Sea has been changing along with the climate, moving from an arctic to a subarctic system. Several fish species have been observed farther north than previously reported and in increasing abundances. We examined one of these fish species, Pacific cod, in the northern Bering Sea (NBS) to assess whether they migrated from another stock in the eastern Bering Sea (EBS), Gulf of Alaska, or Aleutian Islands, or whether they represent a separate population. Genetic analyses using 3,599 single nucleotide polymorphism markers indicated that nonspawning cod collected in August 2017 in the NBS were similar to spawning stocks of cod in the EBS. This result suggests escalating northward movement of the large EBS stock during summer months. Whether the cod observed in the NBS migrate south during winter to spawn or remain in the NBS as a sink population is unknown.

## 1 INTRODUCTION

Several recent studies have confirmed the prediction that increases in water temperature driven by climate change can cause range shifts of marine species toward higher latitudes and contraction at lower latitudes (Booth, Bond, & Macreadie, 2011; Pinsky, Worm, Fogarty, Sarmiento, & Levin, 2013; Sunday, Bates, & Dulvy, 2012). As productive, commercially fished species move northward, understanding the effect of changes in climate on species range shifts into higher latitudes and movement into marginal habitat will be particularly challenging. Even understanding whether habitat is marginal is difficult as conditions shift under climate change. Nevertheless, understanding changes in distribution is important for their conservation through dynamic fisheries management, to engage in decision‐making that is based on the best estimates of stock dynamics and spatial distribution.

Populations in marginal habitats are expected to be less abundant than those in core habitats, and survival and reproductive rates may be lower (Kawecki, 2008; MacCall, 1990; Sexton et al., 2016). Newly established marginal populations are less well adapted, with low reproduction and survival, and must be maintained by migration from the core (Kawecki, 2008). Marginal populations may then adapt to local conditions but are still prone to local extinctions and can act as demographic sinks (Kawecki, 2008). If the new population represents a founder effect, in which a small number of colonizers establish a new self‐sustaining population, the resulting population may exhibit reduced genetic diversity as well as genetic similarity to its population of origin. Adaptation to marginal habitat may also be countered by high immigration from core populations, an effect known as migration swamping (Kirkpatrick & Barton, 1997; Kawecki, 2008; Sexton et al., 2016). If dispersal is high, gene flow may prevent differentiation between the core and marginal populations. In this case, both marginal and core populations could evolve toward intermediate trait values, not optimized for either habitat, with loss of fitness in the core population (Kawecki, 2008).

Changing climate conditions have recently been observed in the Bering Sea and Gulf of Alaska (GOA), and range shifts of several commercially important fish species have been observed in the Bering Sea (Stevenson & Lauth, 2012, 2019). There has also been a change in the Bering Sea from high annual variability in the extent of spring (March–April) sea ice from 1972 to 2000 to prolonged warm intervals with low spring ice extent from 2001 to 2006 followed by more extensive sea ice from 2007 to 2013 (Alabia et al., 2018; Stabeno et al., 2012). Since 2014, the eastern Bering Sea (EBS) has exhibited anomalously warm temperatures (Alabia et al., 2018). As a result, the “cold pool,” water below 2°C that remains along the EBS shelf during the summer following sea ice retreat, has been restricted to the northern parts of the EBS. Most recently, in 2018, the National Marine Fisheries Service (NMFS) survey of the EBS observed the smallest cold pool in the survey history (1982–2018), with only 1% of the total area of the EBS shelf bottom at less than 2°C (Siddon & Zador, 2018; Stabeno et al., 2018). The cold pool is an important factor affecting species distributions; for example, walleye pollock (*Gadus chalcogrammus*), Pacific cod (*Gadus macrocephalus*), and most flatfishes avoid it (Hollowed, Planque, & Loeng, 2013; Sigler et al., 2016; Stevenson & Lauth, 2019). Reductions in marine mammal and benthic prey populations have been observed in the northern Bering Sea (NBS) concurrent with increases in pelagic fish, as the NBS is shifting from arctic to subarctic conditions (Grebmeier et al., 2006).

**Figure 1:**
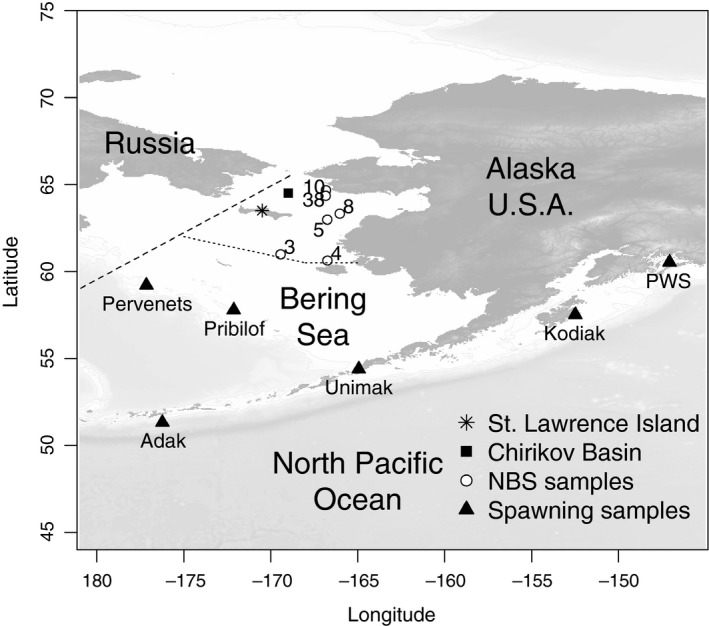
Map showing the study area in the Bering Sea with collection locations: northern Bering Sea (NBS), Pervenets Canyon, Pribilof Islands, Unimak, Adak, Kodiak Island, and Prince William Sound (PWS). The number of cod collected in the NBS is shown next to each collection location. The Russia–U.S. maritime boundary is a dashed line, and the NMFS northern Bering Sea area is north of the dotted line

**Figure 2:**
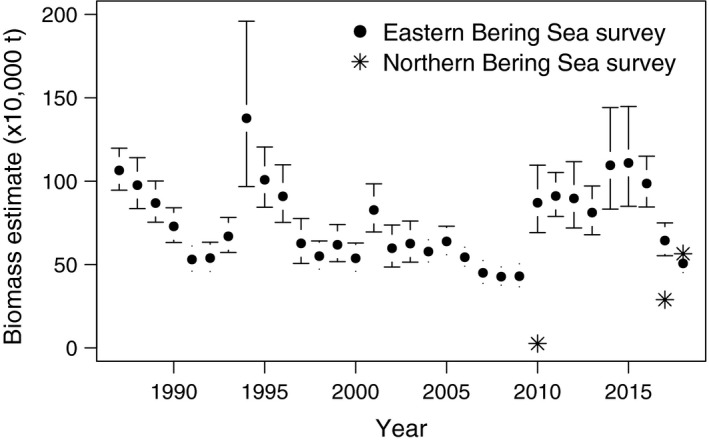
Pacific cod biomass estimates from the eastern Bering Sea (“expanded area”) survey from 1987 to 2018 and the northern Bering Sea survey (2010, 2017, and 2018)

A notable sign of ecosystem change was an increase in the NBS cod biomass during NMFS research surveys conducted in 2017 and 2018. Extensive aggregations of large Pacific cod were recorded to the south of St. Lawrence Island and to the north in Chirikov Basin (Figure 1), yielding biomass estimates of 286,309 tons for the northern region in 2017 (Stevenson & Lauth, 2019) and 564,684 tons in 2018 (Siddon & Zador, 2018). In contrast, the first of the two most recent and comprehensive surveys, conducted in 2010, found very few Pacific cod anywhere north of 60°N latitude, estimating 29,091 tons for the entire northern region, or approximately 3.3% of the biomass for the EBS shelf for that year (Stevenson & Lauth, 2019). Data from previous surveys indicate that the historical range of Pacific cod did not include the northern portion of the Bering Sea; Pacific cod accounted for less than 1% of the total gadid biomass and were encountered “only in trace amounts” in the 1976 and 1979 surveys of Norton Sound and Chirikov Basin (Sample & Wolotira, 1985; Wolotira, Sample, & Morin, 1977). The EBS is considered part of the core habitat for the species, and long‐term average (1980–2018) cod biomass in the EBS is approximately 800,000 t, ten times higher than the Aleutian Islands and three times larger than the GOA (Barbeaux et al., 2017; Schmidt et, 2019). However, the cod biomass measured by the NMFS EBS shelf survey declined 37% between 2016 and 2017, representing the largest decline since the survey began in 1982. Strikingly, the 2017 summer cod biomass in the NBS was equal to 83% of the reduction in biomass in the EBS in the same year and by 2018, summer cod biomass was higher in the NBS than the EBS (Figure 2, Table S2; Thompson, 2018).

The life history of Pacific cod is characterized by seasonal movement between summer feeding and winter spawning locations (Shimada & Kimura, 1994). This seasonal movement coupled with genetic evidence for population structure among spawning groups indicates that natal homing and spawning site fidelity are part of Pacific cod life history, and thus, collections of spawning samples are representative of a population while samples collected during summer migrations may be mixed. Spawning areas have been identified along the Bering Sea shelf, Aleutian Islands, and Gulf of Alaska (Figure 1; Neidetcher, Hurst, Ciannelli, & Logerwell, 2014). It is doubtful that cod have historically spawned in the NBS, since seasonal sea ice typically covers the northern part of the Bering Sea shelf during January–May, even in warm years, and historical surveys observed few cod in the NBS (Sample & Wolotira, 1985; Stabeno et al., 2017; Wolotira et al., 1977).

Shifts in the spatial distribution of commercial species have the potential to disrupt fisheries with challenging implications for sustainable management (Karp et al., 2019; Pinsky et al., 2018). In the Bering Sea, Pacific cod fisheries are rationalized and managed through a complex catch share program involving a diverse suite of fishing sectors (Fina, 2011; Ono et al., 2017; Stram & Evans, 2009). Of particular importance to our study, the northern region of the Bering Sea shelf is closed to commercial trawling, limiting access of one sector of the fleet when the population shifts north. The trawl fishery primarily operates during the fall and winter when fish have returned to the south to spawn. If climate change disrupts or retards this southward migration, fishing opportunities would diminish the catch potential for this portion of the fleet. Likewise, if cod in the northeastern Bering Sea represented a new distinct stock, additional measures to sustainably manage the northern and southern stocks would be necessary as mandated by current US legislation (Magnuson‐Stevens Fishery Conservation and Management Act; Cadrin et al., 2013). However, as in many other marine species, the mechanisms of northward shifts and the status of new northern populations are currently unknown.

The goal of this study was to examine the mechanisms and genetic effects of shifts in the abundance of Pacific cod in the northern portion of its range within the context of our current understanding of range expansions into marginal habitat. This work built upon baseline information (Drinan et al., 2018), which showed that spawning populations of Pacific cod were sufficiently discriminated by SNP loci for successful assignment to population of origin. We used 3,599 single nucleotide polymorphism (SNP) markers obtained through restriction‐site associated DNA sequencing (RADseq) to compare a sample of Pacific cod taken from the NBS during a 2017 research survey to spawning fish from three locations in the EBS and three additional populations throughout their range in Alaska (Figure 1). We tested several hypotheses to explain the origin of increasing abundances of Pacific cod in the NBS, specifically whether the NBS cod stock represents: 1. an isolated pre‐existing population, 2. a new population established via a founder event, 3. a range shift from core habitat elsewhere in the range of Pacific cod, 4. a sink population that is maintained by immigration from core habitat elsewhere in the range of Pacific cod, or 5. an extension of summer feeding migrations. Hypothesis 1 would be consistent with significant genetic differentiation among NBS samples and other known Pacific cod populations, hypothesis 2 would suggest a reduction in relative genetic diversity in NBS cod, while hypotheses 3, 4, and 5 would indicate genetic similarity with the stock of origin. Distinguishing among hypotheses 3, 4, and 5 will require additional research, but the management implications among them are significant. Therefore, results are discussed in the context of other relevant biological information that may provide some further insight and context for the current and future status of the population.

## 2 METHODS

### 2.1 Sample collection

**Figure 3:**
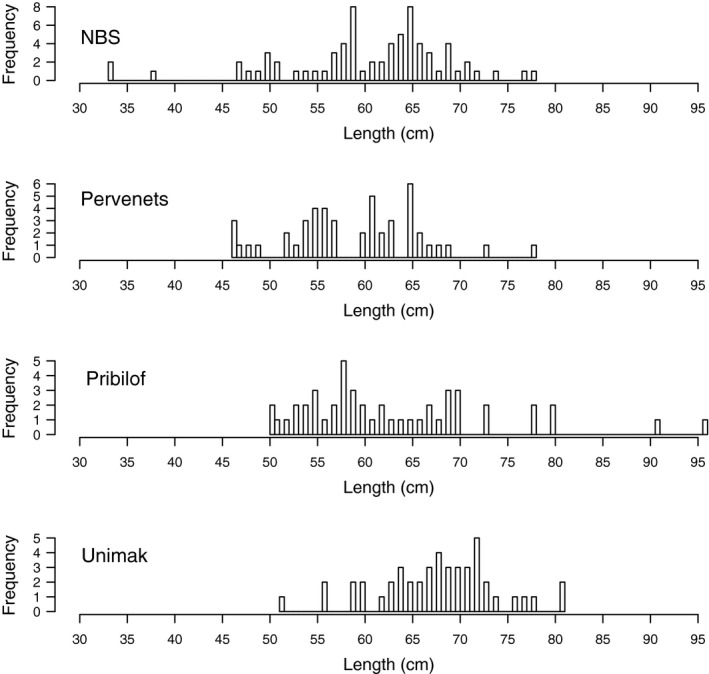
Frequency histogram of fork length (cm) of the 68 fish analyzed from the northern Bering Sea, as well as length frequencies of cod sampled from Pervenets, Pribilof, and Unimak

Fin clips from spawning aggregations in Prince William Sound (March 2012, *n* = 48), near Kodiak Island (March 2003, *n* = 47), and near Adak Island (March 2006, *n* = 49) were collected as described in Drinan et al. (2018). Additional fin clips were collected onboard fishing vessels from spawning aggregations of Pacific cod in Pervenets Canyon (March 2016, *n* = 48), Pribilof Canyon (April 2017, *n* = 48), and Unimak Pass (February 2018, *n* = 48); these fish included spawning, mature, and prespawning individuals (Table 1, Figure 1). Sampling locations were named based on the closest large geographical feature. Lengths were not collected from spawning fish from Adak, Kodiak, or Prince William Sound, as these were collected opportunistically on fishing vessels. Length frequencies for the NBS, Pervenets, Pribilof, and Unimak collections are shown in Figure 3. Fin clips were also collected from nonspawning fish in the NBS during the NMFS bottom trawl survey in August 2017 (Table 1). Previously published sequences (Drinan et al., 2018) were combined with new data for a total of 360 individuals (Table 1).

**Table 1:** Collection locations and dates for samples analyzed in this study

Location	Month/Year	Lat.	Long.	*N*
Adak Island*	Mar. 2006	51°40′N	176°36′W	45(4)
Kodiak Island*	Mar. 2003	57°48′N	152°31′W	45(2)
Prince William Sound*	Mar. 2012	60°32′N	147°4′W	47(1)
Northern Bering Sea	Aug. 4, 2017	60°59′N	169°26′W	3
	Aug. 18, 2017	60°37.8′N	166°44.8′W	4
	Aug. 18, 2017	62°59′N	166°45′W	5
	Aug. 18, 2017	63°19′N	166°02′W	8
	Aug. 23, 2017	64°40′N	166°50′W	10
	Aug. 23, 2017	64°21′N	166°50′W	38
				68(4)
Pervenets Canyon	Mar. 28 2016	59°21′N	177°13′W	48
Pribilof Canyon	Apr. 10 2017	57°47′N	172°8′W	48
Unimak Pass	Feb. 7 2018	54°35′N	165°15′W	47(1)

Note

Samples marked with an asterisk * were sequenced previously (Drinan et al., 2018). *N* represents the number of samples that passed data quality checks, and numbers in parentheses represent the number removed.

### 2.2 RAD sequencing

Fin clips were preserved in 100% nondenatured ethanol. For the NBS collection, Pervenets, Pribilof, and Unimak, RAD libraries were prepared as described in Drinan et al. (2018) and sequenced in 100 bp single‐end reads on three lanes on a HiSeq4000 (Illumina, Inc.) at the University of Oregon Genomics and Cell Characterization Core Facility (GC3F).

### 2.3 STACKS pipeline and data analysis

Data sequenced on the Illumina HiSeq 4000 were downloaded from the GC3F website. Individuals from Prince William Sound, near Kodiak Island, and near Adak Island were previously sequenced on an Illumina HiSeq 2000 (Drinan et al., 2018). Raw reads were filtered and demultiplexed using the STACKS v. 1.44 (Catchen, Hohenlohe, Bassham, Amores, & Cresko, 2013) component program process_radtags with flags (−r −c –q), which rescued barcodes, removed any read with an uncalled base, and discarded reads with low‐quality scores (<90% probability of being correct). Reads were trimmed to 92 bp, based on sequence quality scores using FastQC v.0.11.5 (Andrews, 2010). Reads were aligned to the gadMor2 Atlantic cod genome (Tørresen et al., 2017) using bowtie legacy 1.2 software v.2.3.4.1 (Langmead & Salzberg, 2012), using options (−v 3, −k 2), where −v is the number of mismatches permitted regardless of quality and −k is the number of valid alignments to report. The STACKS pipeline was run with –m 3 in pstacks, −g in cstacks, and –m 3, −r 0.8 in populations using a population map based on collection location. STACKS processing parameters (−m 3 in pstacks and –m 3 in populations) were used to effectively combine datasets run on different platforms, given typically lower stack depth from the HiSeq 2000. Loci with a minor allele frequency (MAF) <0.05 and >0.45 were removed. The loci with MAF 0.45 and higher were typically heterozygous in all individuals and may have been paralogous loci. A single SNP at each locus with the highest MAF was retained for further analysis.

Conformation of genotype proportions to Hardy–Weinberg expectations (HWE) was tested by chi‐squared tests in *pegas* (Paradis, 2010) for each collection separately. Loci were removed if they were not present in at least six of the seven populations, significantly out of HWE following correction for multiple testing by the false discovery rate (FDR) method (Benjamini & Hochberg, 1995), or out of HWE in two or more populations.

Pairs of SNPs displaying linkage disequilibrium within populations were identified with the squared correlation statistic *r* ^2^ using PLINK software v1.90b5.3 (Purcell et al., 2007). Groups of SNPs linked with an *r* ^2^ threshold >0.8 were found using the custom function in R SNPpruner.R (https://github.com/31ingrid/SNPpruner). The 0.8 threshold for *r* ^2^ has been used in previous studies and was intended to balance losing SNPs of interest, if lower, with including physically linked loci, if higher (Jasonowicz, Goetz, Goetz, & Nichols, 2016). Within groups, or clusters of linked SNPs, the linked SNP with the least missing data was retained and the remainder was pruned from the dataset. Further pruning took place in PLINK to identify and remove loci with missing call rates >25% (‐‐geno 0.25) and individuals with missing data >0.3 (‐‐mind 0.3).

We applied OutFLANK v0.2 (Whitlock & Lotterhos, 2015) and BayeScan v2.1 (Foll & Gaggiotti, 2008) to test for the presence of candidate loci under selection in the full dataset (seven populations) and a Bering Sea only dataset (Pervenets, Pribilof, Unimak, and NBS). BayeScan uses differences in allele frequencies between populations to identify loci under selection via a q‐value, a stringent FDR analog of the p‐value. OutFLANK infers the distribution of neutral markers from a trimmed distribution of *F_ST_* values and uses that distribution to detect outliers. BayeScan was run for a total of 100,000 iterations, a burnin of 50,000, and thinning interval 10, for a total sample size of 5,000. The prior odds for the neutral model was set to 100 and *α* = .05 (FDR). OutFLANK was run with left and right trim fractions 0.05, minimum heterozygosity required to include a locus 0.1, and a q‐threshold 0.05. Linkage group and position were taken from the stacks output file sumstats.tsv and aligned with all four GadMor2 annotation files (https://osf.io/4qsdw/) (Tørresen et al., 2017) using the Bioconductor package *GenomicRanges* (Lawrence et al., 2013).

*F*_IS_ and Fisher's exact tests for differentiation were calculated using *genepop* with dememorization of 10,000; 1,000 batches; and 5,000 iterations per batch (Rousset, 2008). The number of alleles per population and expected heterozygosity (*H_e_*) were calculated using *adegenet* (Jombart & Ahmed, 2011). Effective population size was estimated for each population using *NeEstimator* v2.01 with the random mating model, linkage disequilibrium method, parametric 95% confidence intervals, and minimum allele frequency cutoff of 0.05 (Do et al., 2014). *Hierfstat* (Goudet, 2005) was used to calculate rarefied allelic counts and pairwise *F_ST_* (Weir & Cockerham, 1984). Global *F_ST_* and pairwise *F_ST_* with 95% confidence intervals were calculated using *diveRsity* (Keenan, McGinnity, Cross, Crozier, & Prodöhl, 2013). Deviation of each *F_ST_* estimate from zero was estimated by 1,000 random permutations over individuals and over loci, with significance over individuals estimated as the proportion of permuted pairwise *F_ST_* estimates greater than the true *F_ST_*. Significance was calculated by permuting over loci using *StAMPP* (Pembleton, Cogan, & Forster, 2013).

Discriminant analysis of principal components (DAPC) in *adegenet* (Jombart & Ahmed, 2011) was used to visualize spatial relationships in the data. The number of principal components used in the DAPC was determined by comparing reassignment to empirical clusters with that to random clusters using the function *optim.a.score* in *adegenet*. Two DAPC plots were generated, one with all samples and a second with only samples from the Bering Sea.

A mixed stock analysis was performed to identify possible source populations for the NBS collection, with the remaining collections used as reference populations, in *RUBIAS* (Moran & Anderson, 2019). *RUBIAS* is a Bayesian hierarchical genetic stock assignment approach that accounts for population structure among baseline populations (Anderson, Waples, & Kalinowski, 2008). Posterior density curves and 95% credible intervals for mixture proportions were created from MCMC output. A Z‐score was computed from each individual's log‐likelihood and compared with a simulated normal density to test whether individuals in the NBS originated from populations outside the reference set (e.g. from Russia); Z‐scores similar to the normal density provide evidence that the mixture sample came from one of the reference populations. A Kolmogorov–Smirnov test was used to compare the Z‐score with the normal density. The mixture analysis was performed on six reference datasets, each of which included three collections not from the Bering Sea (Adak, Prince William Sound, and Kodiak) as well as follows: (a) EBS individuals (Pervenets, Priblof, and Unimak) combined into a single population; (b) EBS individuals as separate populations; (c) all EBS populations excluded; (d) Pervenets excluded; (e) Pribilof excluded; and (f) Unimak excluded. The accuracy of the individual assignment analysis was evaluated by testing self‐assignment of simulated individuals of known origin. Known simulated proportions for each reporting group were compared with the numbers estimated by *RUBIAS* to test the accuracy of the individual assignment analysis.

## 3 RESULTS

Data processed on the Illumina HiSeq 4000 averaged 3.8 × 10^8^ total sequences per library, 5.4% ambiguous barcode drops, 0.6% low‐quality read drops, and 88.5% retained read rate. Data processed on an Illumina HiSeq 2000 presented lower average scores: 1.9 × 10^8^ total sequences per library, 13.0% ambiguous barcode drops, 8.0% low‐quality read drops, and 53.1% retained read rate. A total of 6,133 loci were retained following initial STACKS v1.44 data processing. Five individuals were removed during the STACKS pipeline due to insufficient data (Table S1).

There were 3,731 loci remaining after selection for loci present in at least six out of seven populations and in HWE by population. Sixty‐six loci were removed due to linkage disequilibrium, and the largest linked cluster consisted of three loci. Seven individuals were removed with more than 30% missing data (Table S1). An additional 66 loci were removed that contained more than 25% missing data over all individuals (geno > 0.25). A total of 3,599 loci and 348 individuals remained in the final dataset, with 2.52% missing data, after data quality checks. The number of individuals per location in the final dataset ranged from 45 to 48, with the exception of the NBS collection, which contained 68 individuals that ranged in fork length from 33 to 78 cm (Table 1, Figure 3).

**Figure 4:**
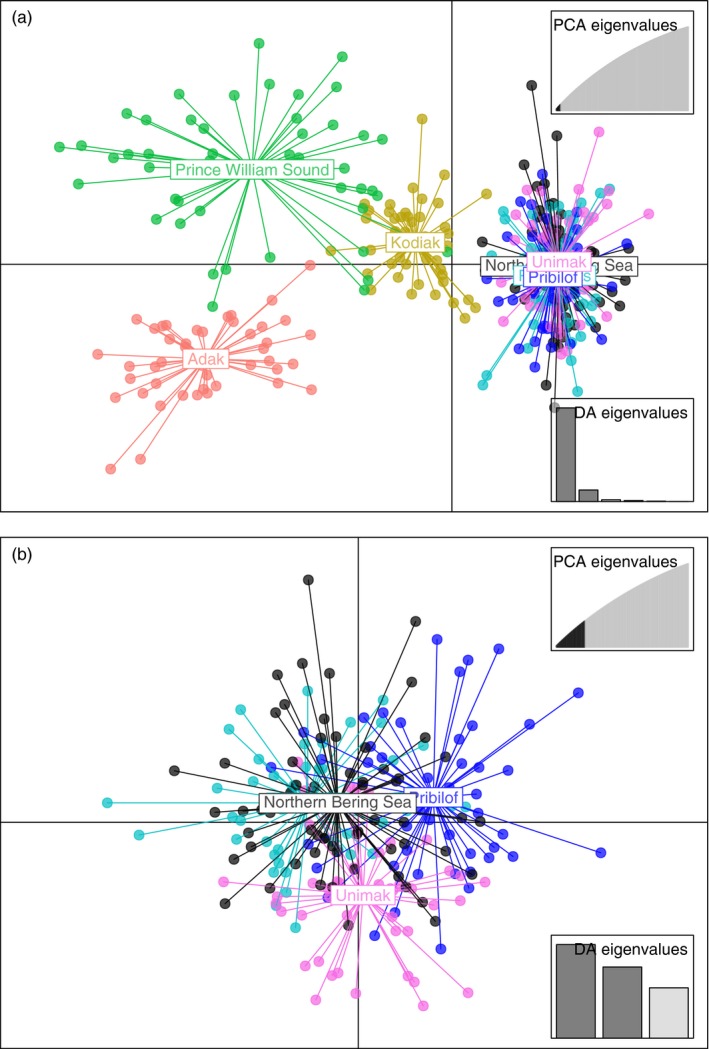
(a) DAPC scatterplot of samples from the northern Bering Sea, Pervenets Canyon, Pribilof Islands, Unimak Pass, Adak, Kodiak Island, and Prince William Sound. Note: Samples from the northern Bering Sea, Pervenets Canyon, Pribilof Islands, and Unimak Pass overlap. Insets indicate the number of principal components retained (PC = 12). (b) DAPC scatterplot of samples from the Bering Sea only (northern Bering Sea, Pervenets, Pribilof, and Unimak), PC = 46. Note: Pervenets is in teal green

*F*_IS_ was generally low and positive, indicating a heterozygote deficiency, which can be due to inbreeding or allelic dropout (Table 2). Global *F_ST_* over all samples was 0.0059 (0.0044, 0.0078) and pairwise *F_ST_* ranged from 0.0002 to 0.0142 across all comparisons (Table 3). DAPC was optimized for all samples using 12 principal components and distinguished four clusters: Prince William Sound, Adak, Kodiak, and the eastern Bering Sea (Figure 4a). A DAPC limited to Bering Sea samples showed no further structure (Figure 4b) with 46 principal components.

**Table 2:** Summary statistics for each collection location: number of individuals analyzed (*N*), number of alleles (*A*), the rarefied allelic richness (*A_R_*), the inbreeding statistic *F*_IS_, expected heterozygosity *H_e_*, and effective population size with 95% confidence intervals

Sampling location	*N*	*A*	*A_R_*	*F*_IS_	*H_e_*	*N_e_* (95% CI)
Adak	45	7,166	1.9817	−0.0160	0.287	1,691 (1,432; 2,064)
Kodiak	45	7,165	1.9768	0.0222	0.281	2,868 (2,223; 4,036)
Prince William Sd.	47	7,186	1.9852	0.0079	0.287	3,746 (2,814; 5,594)
*N*. Bering Sea	68	7,142	1.9731	0.0225	0.285	15,130 (8,147; 104,559)
Pervenets	48	7,133	1.9714	0.0203	0.283	6,198 (4,095; 12,709)
Pribilof	48	7,126	1.9725	0.0165	0.286	22,432 (7,789; ∞)
Unimak	47	7,135	1.9717	0.0150	0.283	∞ (63,413; ∞)

**Table 3:** Below diagonal: pairwise *F_ST_* estimates for all sample locations: Adak, Kodiak, Prince William Sound (PWS), northern Bering Sea (NBS), Pervenets, Pribilof, and Unimak

	Adak	Kodiak	PWS	NBS	Pervenets	Pribilof	Unimak
Adak		0.0930	***	***	0.000	0.000	0.000
Kodiak	0.0096** (0)		0.000	0.166	0.406	0.412	0.911
PWS	0.0074** (0)	0.0051** (0)		***	***	***	0.855
NBS	0.0136** (0)	0.0033** (0)	0.0100**(0)		1.000	1.000	1.000
Pervenets	0.0136** (0)	0.0037** (0)	0.0102** (0)	0.0002 (0.197)		1.000	1.000
Pribilof	0.0137**(0)	0.0033** (0)	0.0103**(0)	0.0002 (0.202)	0.0007* (0.016)		1.000
Unimak	0.0142** (0)	0.0032** (0)	0.0095** (0)	0.0002 (0.279)	0.0008* (0.011)	0.0006* (0.021)	

Note

Significance estimates for *F_ST_* bootstrapped over loci are presented as asterisks (** = *p* < .001, * = *p* < .05), and *p*‐values are shown in parentheses for bootstraps over individuals. Above diagonal: *p*‐values from Fisher's exact tests for differentiation over all loci; *** indicates highly significant.

Of the 3,599 loci in the final dataset, 3,543 aligned to linkage groups in the Atlantic cod genome (GadMor2) and the remainder aligned to genomic scaffolds. The loci were represented relatively evenly across all 23 linkage groups (LG), ranging from 81 on LG17 and 192 on LG14 (Table S3). OutFLANK identified 218 outlier loci in the full dataset and four in the Bering Sea dataset, while BayeScan identified 90 outlier loci in the full dataset and one in the Bering Sea dataset. All but one of the candidate loci for selection identified by BayeScan in the full dataset was also identified with OutFLANK. These 89 loci mapped to a limited number of linkage groups: LG2, 6, 8, 9, 11, 14, and 15, suggesting that some linkage groups may contain more genes under selection than others (Table S3). One of these SNPs, with *F_ST_* = 0.11245 and *α* = 0, mapped to LG9, position 24,173,727, was located within the *zona pellucida* subunit 3 gene sequence. Other gene functions were not identified, but several of the highest *F_ST_* loci were found in similar locations along LG6. A single SNP locus was identified to be a candidate for selection with BayeScan and OutFLANK in the Bering Sea dataset. This mapped to LG6, position 11,812,308, but gene function was not annotated. Alpha was positive for all SNPs identified as outliers, indicating diversifying selection rather than balancing. BayeScan results are shown visually for the full and Bering Sea datasets, with log_10_(*q*‐value) plotted versus *F_ST_*, and a vertical line depicting the threshold level for significance (Figure S1).

Effective population size estimates were generally large and ranged from 1,691 in the Adak population to over 5,000 in samples from the Bering Sea, although results are likely imprecise as some values could not be estimated (Table 2). The Unimak effective population size could not be estimated (expressed as infinity), although the lower bound of the 95% confidence interval was 63,413. Overall, estimates of effective population sizes of islands and inlets tended to be smaller than shelf populations. The range of rarefied allelic richness was narrow, 1.971–1.985, and the NBS collection had the highest allelic richness out of all EBS populations.

Significance estimates obtained by bootstrapping *F_ST_* over loci and over individuals were comparable (Table 3, below diagonal). All pairwise *F_ST_* estimates except those between the NBS and EBS were significant over loci and individuals, providing support for significant differentiation among all spawning populations (*p* < .001). Comparison of the northern Bering Sea collections to Pervenets, Pribilof, and Unimak were not significant (*p* > .05), while comparisons among Bering Sea collections were significant at *p* < .05 (Table 3). Spawning collections from the Bering Sea were genetically distinct, but could not be distinguished from the NBS collection when measured by *F_ST_*. This result was consistent with the NBS collection being a mixture of several Bering Sea spawning populations.

Genetic differentiation among collections measured by Fisher's exact test for differentiation and *F_ST_* were generally congruent with a few exceptions. Fisher's exact tests were more conservative than *F_ST_* in differentiating among populations (Table 3, above diagonal). The NBS collection was not genetically distinguishable from the EBS populations (Pervenets, Pribilof, and Unimak) under *F_ST_* or Fisher's exact tests (Table 3); we cannot reject the null hypothesis that alleles from NBS and EBS populations were drawn from the same distribution. However, unlike *F_ST_*, Fisher's exact tests among the Bering Sea collections (Pervenets vs. Pribilof, Pervenets vs. Unimak, and Pribilof vs. Unimak) did not show significant differences among collections.

**Figure 5:**
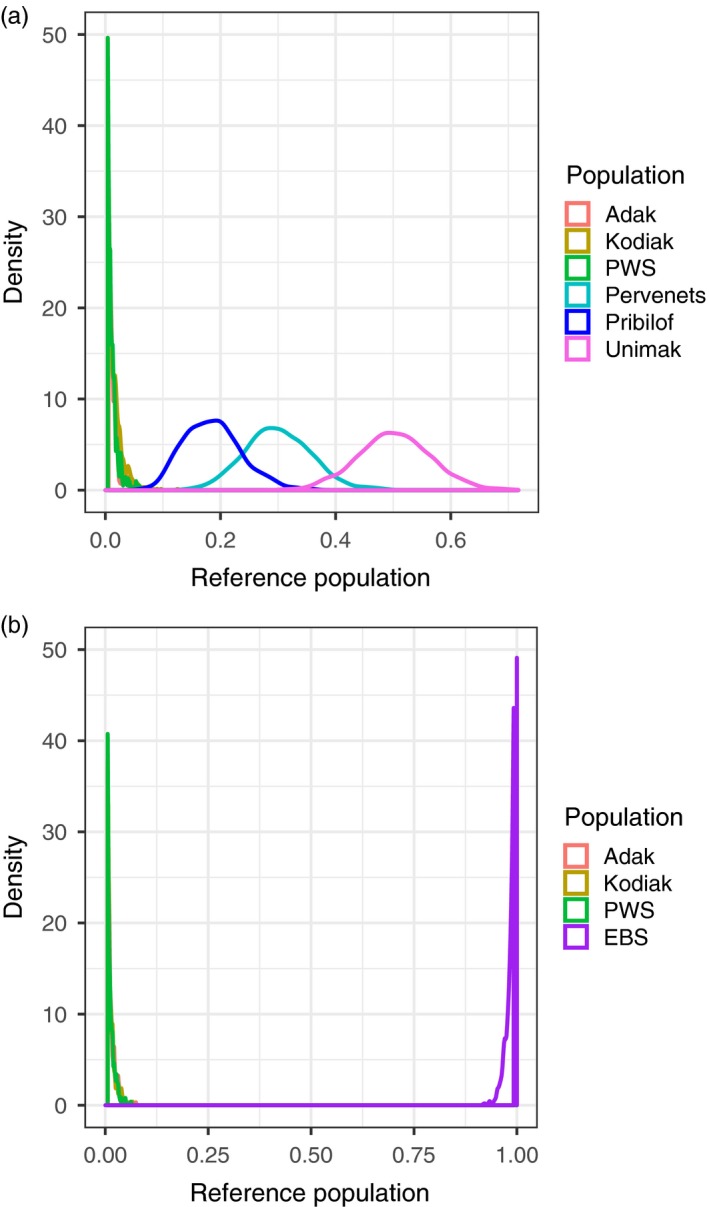
Posterior density of the probability of assignment of the northern Bering Sea sample to reference spawning populations, labeled as “Population” in the legend. In panel (a), Bering Sea reference populations (Pervenets, Pribilof, and Unimak) were applied separately and in panel (b), Bering Sea reference populations were combined. Note: PWS = Prince William Sound

The estimated posterior density for the *RUBIAS* mixture proportion of the NBS individuals was over 98% (0.957, 1.000) EBS when individuals from Pervenets, Pribilof, and Unimak were combined into a single EBS reference population (Figure 5a, Table 4a). When Pervenets, Pribilof, and Unimak were treated as separate reference populations, the mixture proportions were 30% Pervenets, 19% Pribilof, and 50% Unimak (Figure 5b), with <1% for populations outside of the EBS (Table 4b).

**Table 4:** Probability (*p*) of assignment of the northern Bering Sea sample to reference populations, with low and high 95% credible intervals (low CI, high CI), for (a) eastern Bering Sea samples combined as a single “EBS” population, and (b) EBS samples used as separate reference populations

Assignment to reference population	*p*	Low CI	High CI
(a)			
Adak	.003	5 × 10^–9^	0.025
Kodiak	.004	7 × 10^–9^	0.025
Prince William Sound	.004	2 × 10^–9^	0.024
Eastern Bering Sea	.989	0.957	1.000
(b)			
Adak	.003	4 × 10^–13^	0.022
Kodiak	.007	3 × 10^–11^	0.039
Prince William Sound	.002	1 × 10^–11^	0.018
Pervenets	.302	0.2	0.421
Pribilof	.186	0.1	0.290
Unimak	.500	0.4	0.620

**Figure 6:**
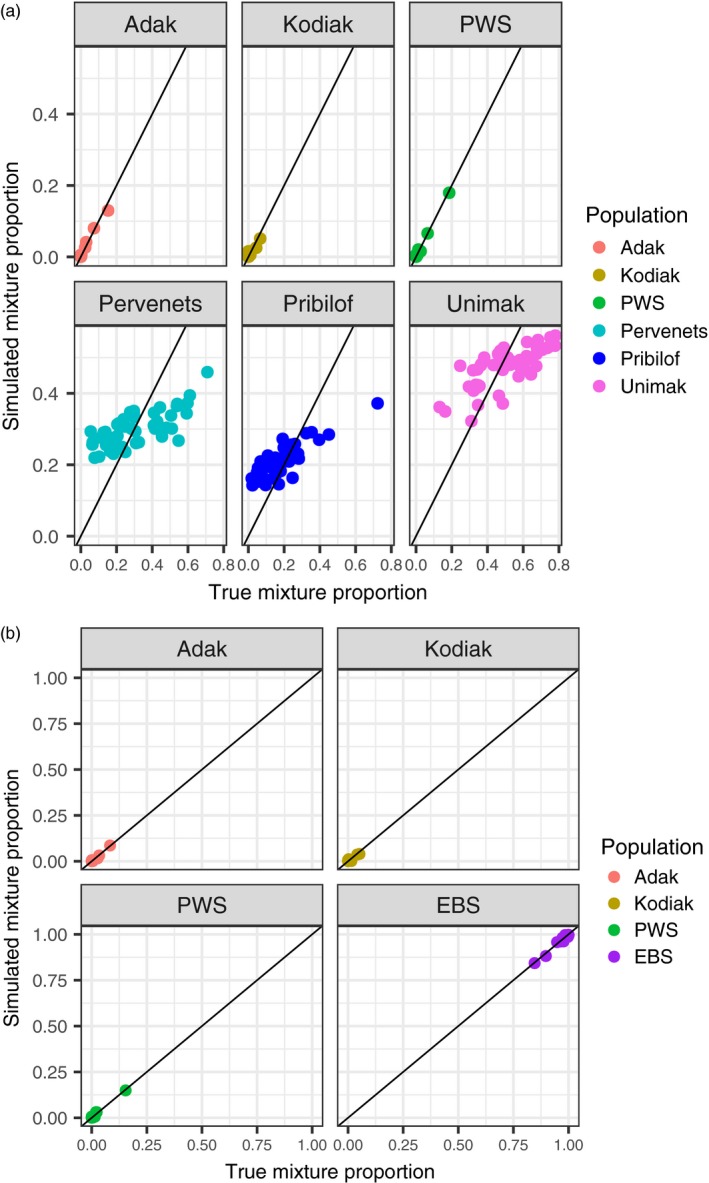
Comparison of true mixture proportion and simulation mixture contributions for each of the four reporting groups for (a) a reference dataset with eastern Bering Sea reference populations considered separately and (b) the reference dataset with Pervenets, Pribilof, and Unimak combined into a single eastern Bering Sea group

Comparison of the Z‐scores to the normal distribution provided information on whether the NBS collection came from a population not represented by reference populations. The Kolmogorov–Smirnov test rejected the null hypothesis that the log‐likelihood Z‐score came from a normal distribution when all EBS samples were excluded from the reference but not otherwise (Table 5). Removal of Pervenets, Pribilof, or Unimak from the reference dataset did not significantly change the Z‐score, even though each had a high probability of assignment from the NBS (Table 5). Simulations indicated that mixture proportions were highly accurate for all reference groups when EBS collections were combined (Figure 6a). When considered separately, there was a definitive loss of accuracy in the mixture proportions of Pervenets, Pribilof, and Unimak populations (Figure 6b), which was likely associated with low levels of differentiation among those populations.

**Table 5:** The *p*‐values associated with the null hypothesis that the total log‐likelihood was drawn from the reference distribution, or the simulated normal density, based on the Kolmogorov–Smirnov test, for several reference population scenarios of the mixture analysis

Scenario	K‐S *p*‐value
EBS individuals combined into a single reference population	0.2014
EBS individuals incorporated as separate reference populations	0.7828
All EBS individuals excluded from reference population	3.91e−10
Pervenets excluded from reference population	0.3247
Pribilof excluded from reference population	0.4664
Unimak excluded from reference population	0.5700

Note

Similarity among total log‐likelihood and simulated normal densities (significant test scores) are an indication of whether northern Bering Sea collection could have come from the reference populations.

## 4 DISCUSSION

We found evidence for large‐scale summer redistribution of Pacific cod from their historical range in the southern EBS to ~1,000 km north during anomalously warm conditions between 2010 and 2017. Movement appeared to be most likely from spawning populations in the EBS core habitat into the NBS, which has historically been marginal habitat as is evidenced by low encounter rates for surveys conducted in the region before 2017 (Bakkala et al., 1992; Bakkala, Traynor, Teshima, Shimada, & Yamaguchi, 1985; Goddard & Zimmermann, 1993; Sample & Wolotira, 1985; Stevenson & Lauth, 2012, 2019; Walters et al., 1988; Wolotira et al., 1977; Wolotira, Sample, Noel, & Iten, 1993). Individual cod collected from the NBS in August of 2017 assigned to EBS spawning populations with high levels of certainty (>98%) and not to the Aleutian Islands or the GOA (Figure 5; Table 4). This indicated support for hypotheses 3, 4, and 5; the movement into the NBS represented either a large‐scale redistribution (hypothesis 3), a sink population (hypothesis 4) from EBS cod spawning populations, or an extension of the northward feeding migration of the EBS stock (hypothesis 5). It is difficult to distinguish among hypotheses 3, 4, and 5 without further analyses, but a feeding migration is the simplest and most consistent with known cod life history. Pacific cod sampled in the NBS had above average condition in 2018, and anecdotal observations indicated their stomachs were full of snow crab, *Chionoecetes opilio* (Siddon & Zador, 2018). The northern Bering Sea may represent a new location for summer feeding migrations of EBS cod in years with a diminished cold pool. If cod migrate northward to feed during the summer, they may either undergo long migrations back to their spawning location of origin (hypothesis 5) or perhaps overwinter in the NBS if conditions allow (hypotheses 3 or 4).

It is possible that the NBS collection, or some portion thereof, could have originated from Russian waters or another unsampled location, if that population were genetically similar to the reference populations from the EBS. Pacific cod spawn along the Bering Sea shelf, and fishing takes place along the shelf on both sides of the Russia—U.S. maritime boundary during spawning season. It is unlikely that cod in Russian water adjacent to the Pervenets sampling location are strongly genetically distinct from other EBS cod, but this has not been tested. Nonetheless, Pervenets, Pribilof, and Unimak represent the largest and most geographically proximate known spawning areas in the EBS and are therefore the most likely sources of the NBS collection (Neidetcher et al., 2014).

Results indicate against hypothesis 1, a founder event, and hypothesis 2, that large numbers of cod in the NBS originated from a pre‐existing population in the region. Genetic results did not provide evidence that cod observed in the NBS were genetically distinct from populations of cod in the EBS (Tables 2 and 3). The level of allelic richness was not lower in the NBS collection than any of the EBS populations, which would be expected in the case of a founder effect. Rather, slightly higher allelic richness in the NBS may indicate that it represents a mixture of other stocks, a reasonable assumption considering that stocks of cod likely mix during the summer feeding season (Table 2). Further, surveys indicated very low abundance of Pacific cod in the NBS prior to 2010. The length–frequency distribution of cod collected in the NBS was also similar to that of the EBS population, although slightly larger (Stevenson & Lauth, 2019), which could be due to better feeding conditions or if cod preferentially undertake more distant summer migrations as they grow.

Our results provide strong evidence of large‐scale northward movement of eastern Bering Sea Pacific cod into the NBS during summer months. There are significant management implications based on whether the entire life history of the NBS cod has shifted northward (hypothesis 3), whether it represents a sink population with its source the EBS stock (hypothesis 4), or simply an extension of summer feeding migrations (hypothesis 5). Under a distributional shift (hypothesis 3), spawning activity would also shift north, and roughly half of the fishing takes place on spawning stocks; whereas under hypothesis 4 or 5, the spawning stock would remain along the EBS shelf. Almost no knowledge exists on winter (spawning) distributions, as there is no winter survey and no fishing in the NBS.

Oceanographic evidence provides a mechanism for northward transport of juveniles. It is unknown why younger fish would move northward, but it has been shown that young fish follow older fish to learn migration routes (Dodson, 1988). The smallest fish collected from spawning populations was 46 cm from Pervenets and could have been a maturing 3 or 4 years old, as 50% maturity is estimated at 4.8 years in EBS cod (Figure 3, Thompson, 2018). The smallest fish sampled in the Northern Bering Sea were 33 cm and 2 years old, likely too small to swim 600–1,000 km if they originated along the Bering Sea shelf (Figure 3). There is evidence to suggest that larval fish can position themselves in the water column to take advantage of current patterns, suggesting a possible mechanism for northward movement of juvenile cod. Juvenile walleye pollock use a northward baroclinic flow along the 100 and 200 m isobaths of the middle and outer Bering Sea shelf (Duffy‐Anderson et al., 2017; Hurst et al., 2009; Stabeno, Danielson, Kachel, Kachel, & Mordy, 2016). More research is needed to understand under which conditions cod juveniles may use currents for northward transport.

Evidence based on fish condition, recruitment, and catch may inform the current dynamics of the stock in the Bering Sea. Anomalously high catch per unit effort (CPUE) in the March 2018 EBS winter trawl fishery may be interpreted as evidence that spawners return to the eastern Bering Sea, or it may represent a decline in spawning activity. High CPUE can be associated with low biomass (hyperstability) if a species exhibits hyperaggregation. Normalized CPUE in March 2018 was more than double1CPUE was 5.703 kg per 1,000 hooks, with all months for 1991–2018 normalized to a mean of one (Thompson, 2018). any other CPUE in the March trawl fishery since 1991 (Thompson, 2018). Hyperaggregation occurred in Atlantic cod in the Bonavista Corridor off Newfoundland, Canada, where the density of cod from 1990 to 1993 was fourfold that of the 1980s even though abundance had declined fivefold (Rose & Kulka, 1999). While hyperaggregation has been documented in Atlantic cod and other fish species (Erisman et al., 2011; Neuenhoff et al., 2018), it has not yet been evaluated in Pacific cod. Length–weight residuals, used as a proxy for fish condition, have declined in Pacific cod in the EBS since 2003 but increased in the NBS in 2018 relative to the EBS population (Siddon & Zador, 2018), indicating that NBS summer feeding is favorable for cod. Although the spawning stock was large in 2018, estimated annual recruitment has been below average since 2013 (Thompson, 2018). Anomalously low recruitment may be an indication of a disruption in typical spawning behavior or a reduced spawning stock, although estimates of total spawning biomass have appeared relatively stable since 1990s (Figure 1.21, Thompson, 2018). Together, this evidence points to reduced recruitment or reduced spawning activity in the EBS, but high‐quality foraging in the NBS.

Significant stock status changes, likely coupled to environmental change, have occurred recently in other parts of the range of Pacific cod, as well. In the GOA, Pacific cod encountered a different fate than Bering Sea cod during the recent warm period. During 2016–2017, warmer temperatures led to increased metabolic demands in Pacific cod, which may have exceeded energetic consumption and resulted in lower body condition (Barbeaux et al., 2017). Indeed, this elevated mortality was likely the cause of a 58% decline in Pacific cod biomass in the GOA estimated from NMFS biomass trawl survey data in 2017 relative to 2016, the lowest estimate from the time series (1984–2017) of standardized surveys by more than half (Barbeaux et al., 2017). In addition, at the southern end of their range in Puget Sound, WA, abundance declined as predicted in a range contraction typical for ectotherms in general (Sunday et al., 2012). Catches of cod in Puget Sound averaged approximately 2 million pounds from 1958 to 1967 (Alderdice & Forrester, 1971). Today, however, few cod currently reside in Puget Sound, and abundance estimates from WDFW trawl surveys are considered unreliable due to the infrequency of cod encounters within most Puget Sound sub‐basins (Pacunski, R., WDFW, pers. comm.).

Several tangential results are worth mentioning that provide potentially useful management information. Significant *F_ST_* values among EBS shelf populations were present even though the magnitude of *F_ST_* was too small to distinguish among those populations using assignment testing. Fisher's exact test has been shown to have low statistical power for SNPs; therefore, the significant levels determined using pairwise *F_ST_* are considered more reliable (Ryman et al., 2006). The significant pairwise *F_ST_* estimates among EBS populations (Pervenets, Pribilof, and Unimak) warrant further research on the level of genetic differentiation among those populations, perhaps using a more powerful dataset, such as whole‐genome sequencing. A lack of differentiation among the NBS collection and Bering Sea spawning populations provides support that those fish represented a mixture of Bering Sea populations. Overall, effective population size estimates are likely inaccurate, and larger sample sizes are likely required to obtain more precise estimates (Marandel et al., 2019). Even so, effective population sizes support independent population size estimates that indicate the Bering Sea stock is larger than either the Gulf of Alaska or the Aleutian Islands. The data used in this study consisted of primarily selectively neutral loci, with roughly 89 loci in the full dataset identified as candidates for diversifying selection. Assignment testing is not commonly applied to marine populations, which are typified by low levels of *F_ST_*. Incorporating a combination of neutral SNPs and SNPs under diversifying selection is a useful technique to improve assignment success, as in Drinan et al. (2018). The dataset used here included many of the same loci in that study, including one SNP found within the *zona pellucida* subunit 3 coding sequence.

The results of this study are directly relevant to the management of this valuable resource underscoring the growing importance of genetics as a tool for sustainable management in a changing climate. Our study supports the hypothesis that climate change will extend the range for many subarctic species including Pacific cod (Cheung, Reygondeau, & Frölicher, 2016). However, our study does not support the hypothesis that climate‐induced shifts in suitable habitat will also result in increased catch potential. If the low recruitment trend is not abated, the stock and the future catch potential will decline despite the expansion of suitable habitat. The findings supported continued single stock management in the eastern Bering Sea, although observations of cod along border of the United States and Russia suggests that transboundary management agreements may be necessary for the future (Pinsky et al., 2018). Results also emphasize the need for ecosystem‐based fisheries management as a holistic approach that encompasses all interactions within the ecosystem rather than a single‐species approach. In addition to genetics, tagging studies and continued research surveys are essential for understanding how a shifting climate will influence the extent of postspawning migrations in existing populations (e.g. from the EBS) or whether cod will ultimately colonize new spawning areas in the NBS.

## CONFLICT OF INTEREST

None declared.

## Data Availability

The data that support the findings of this study are available in the Sequence Read Archive (SRA), which is accessible from National Center for Biotechnology Information (NCBI) at http://www.ncbi.nlm.nih.gov/bioproject/558810, BioProject ID PRJNA558810 (Spies et al., 2019).
