# Supplementary material for: Genetic evidence of a northward range expansion in the eastern Bering Sea stock of Pacific cod
Source: Evol Appl. 2019 Oct 18;13(2):362–75. doi: 10.1111/eva.12874 (PMC6976961; doi:10.1111/eva.12874)
Supplement: Supplementary file 1 [file EVA-13-362-s001.docx]

**Appendix S1**

Table S1. Individuals removed due to >30% missing data, using the –mind .3 option in PLINK. Individuals with asterisk were removed during the STACKS pipeline.

| Individuals removed |
| --- |
| AD06_002 |
| AD06_022 |
| AD06_031 |
| AD06_034 |
| KOD03_087 |
| KOD03_092 |
| PWS12_078  NBS_2017_26 (H24)*  NBS_2017_28 (H24)*  NBS_2017_52 (H45)*  NBS_2017_75 (H45)*  Unimak_2018_49* |

Table S2. Pacific cod biomass and standard error estimates from the eastern Bering Sea “extended” survey area (1987-2018) and the northern Bering Sea survey (2010, 2017, 2018).

|  | Eastern Bering Sea | | Northern Bering Sea | |
| --- | --- | --- | --- | --- |
| Year | Biomass | Standard error | Biomass | Standard error |
| 1987 | 1,064,600 | 63,998 |  |  |
| 1988 | 976,152 | 77,490 |  |  |
| 1989 | 868,804 | 62,883 |  |  |
| 1990 | 728,996 | 52,790 |  |  |
| 1991 | 530,488 | 38,367 |  |  |
| 1992 | 538,862 | 44,674 |  |  |
| 1993 | 669,305 | 53,151 |  |  |
| 1994 | 1,377,095 | 247,764 |  |  |
| 1995 | 1,008,293 | 91,637 |  |  |
| 1996 | 909,133 | 87,574 |  |  |
| 1997 | 627,151 | 68,411 |  |  |
| 1998 | 550,504 | 43,013 |  |  |
| 1999 | 618,679 | 56,577 |  |  |
| 2000 | 537,563 | 43,172 |  |  |
| 2001 | 827,176 | 73,336 |  |  |
| 2002 | 597,943 | 63,845 |  |  |
| 2003 | 625,659 | 62,403 |  |  |
| 2004 | 578,064 | 33,833 |  |  |
| 2005 | 638,764 | 43,470 |  |  |
| 2006 | 544,035 | 28,965 |  |  |
| 2007 | 450,337 | 35,084 |  |  |
| 2008 | 427,503 | 27,847 |  |  |
| 2009 | 430,084 | 35,061 |  |  |
| 2010 | 870,639 | 102,350 | 26,140 | 6,516 |
| 2011 | 911,082 | 66,919 |  |  |
| 2012 | 896,401 | 100,487 |  |  |
| 2013 | 811,667 | 74,203 |  |  |
| 2014 | 1,095,270 | 153,347 |  |  |
| 2015 | 1,109,115 | 150,994 |  |  |
| 2016 | 986,013 | 77,381 |  |  |
| 2017 | 643,953 | 49,976 | 289,264 | 37,034 |
| 2018 | 506,943 | 29,419 | 564,684 | 127,264 |

Table S3. Genomic location on the Atlantic cod linkage map GadMor2 of all 3,599 loci used in this study, 89 outlier SNPs from the full datset, and one outlier SNP from the Bering Sea datset.

| Linkage group | All loci | Full datset outliers | Bering Sea outlier |
| --- | --- | --- | --- |
| LG01 | 185 | 0 | 0 |
| LG02 | 144 | 17 | 0 |
| LG03 | 175 | 0 | 0 |
| LG04 | 186 | 0 | 0 |
| LG05 | 146 | 0 | 0 |
| LG06 | 170 | 30 | 1 |
| LG07 | 179 | 0 | 0 |
| LG08 | 152 | 31 | 0 |
| LG09 | 158 | 5 | 0 |
| LG10 | 133 | 0 | 0 |
| LG11 | 187 | 3 | 0 |
| LG12 | 160 | 0 | 0 |
| LG13 | 157 | 0 | 0 |
| LG14 | 192 | 2 | 0 |
| LG15 | 168 | 1 | 0 |
| LG16 | 167 | 0 | 0 |
| LG17 | 81 | 0 | 0 |
| LG18 | 157 | 0 | 0 |
| LG19 | 140 | 0 | 0 |
| LG20 | 126 | 0 | 0 |
| LG21 | 132 | 0 | 0 |
| LG22 | 110 | 0 | 0 |
| LG23 | 138 | 0 | 0 |

Figure S1. BayeScan results: log_10_(q-value) vs. *F_ST_* based on the full dataset (panel a.), and the Bering Sea dataset (Pervenets, Pribilof, Unimak, and the NBS collection, panel b.). The vertical line represents the threshold FDR value of 0.05.

| a.  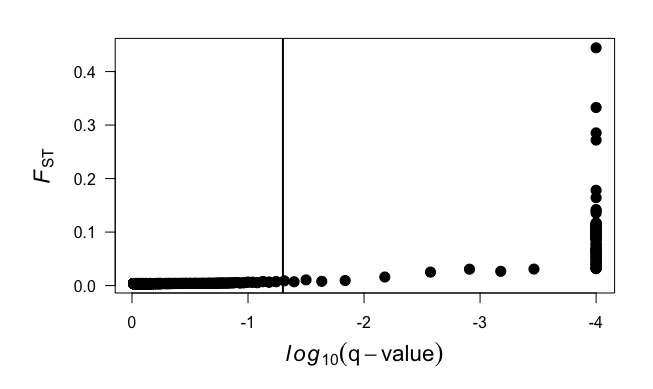 |
| --- |
| b.  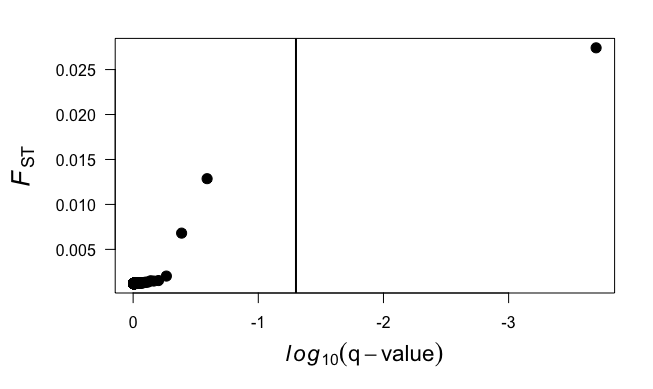 |
